# Co-movements, option pricing and risk management: an application to WTI versus Brent spread options

**DOI:** 10.1007/s10479-022-05059-7

**Published:** 2022-11-14

**Authors:** Domenico De Giovanni, Arturo Leccadito, Debora Loccisano

**Affiliations:** 1 Department of Economics, Statistics and Finance, University of Calabria, Arcavacata, Italy; 2 Department of Economics, Carleton University, Ottawa, Canada; 3 LFIN/LIDAM, UCLouvain, Voie du Roman Pays 34, Louvain la neuve, 1348 Belgium

**Keywords:** Contagion, Spread options, Co-moments, Value at risk, Expected Shortfall

## Abstract

Co-moments of asset returns play a major role in financial contagion during crises. We study the properties of a particular specification of the generalized bivariate normal distribution which allows for co-volatility and co-skewness. With this probability distribution, formulae for single-name and exchange options can be evaluated quickly since they are based on one-dimensional integrals. We provide a very precise approximation formula for spread option prices and derive the corresponding greeks. We perform a day-to-day re-estimation of the probability distribution on a dataset of WTI vs Brent spread options, showing the ability of this specification to capture the salient empirical features observed in the market. Finally, we show the impact of co-movements on portfolio risk management.

## Introduction

High-order co-moments play an important role in the literature on financial contagion, indeed, they are used to identify channels of contagion among asset returns.^1^ Fry et al. (2010) extend correlation-based tests of contagion [see, for instance, Forbes and Rigobon (2002)] and develop statistical tests based on co-moments using a generalized normal distribution. These tests are generalized in Fry-McKibbin and Hsiao (2018) and Fry-McKibbin et al. (2018). Fry-McKibbin et al. (2014) perform an extensive set of numerical experiments to show how option prices and hedging strategies are affected by co-moments. While the papers above focus on tests based on single-channel contagion, more recently Hsiao and Morley (2022) formulate tests based on multiple channels, which establish contagion by identifying changes in co-moments. The authors apply their newly-developed statistical tests to four events in which financial crises originated in one country have spread through the world.

The literature offers other approaches that allow to capture financial contagion in the context of option pricing, one of which consists of the use of mutually self-exciting jump processes (Aït-Sahalia et al., 2014; Aït-Sahalia et al., 2015; Aït-Sahalia & Hurd, 2015; Kokholm, 2016). In the context of single-name options, Melick and Thomas (1997) offer a method and a new model based on mixture of lognormals to estimate the option-based risk neutral density.

Co-moments have also a key role in the pricing of spread options. This is well documented in Fry-McKibbin et al. (2014), where the authors show the effects of high-order co-moments on spread option prices. The literature that studies spread options offers a variety of models and numerical techniques (see Carmona and Durrleman 2003, for a review). Margrabe (1978) provides the formula to price exchange options, a special case in which the exercise price is null. Apart from this case, no closed formula exists, and one must resort to numerical techniques. In this context, two approaches stand out. The first approach is the two-dimensional Fourier transform method of Hurd and Zhou (2010), which is exact, but requires a bivariate numerical integration. The second is the one-dimensional approximation of Caldana and Fusai (2013), which generalizes the lower bound of Bjerksund and Stensland (2014) (improving the approximation formula of Kirk, 1995), valid in the two-dimensional lognormal setup, for any model for which the joint characteristic function is available in closed form. This method requires only a one-dimensional Fourier inversion and provides a very accurate approximation of the true price. From the modeling side, the literature offers a variety of approaches. Huang and Kou (2006) and Cheang and Chiarella (2011) are based on jump diffusion processes. Subordinated Levy processes are used in Ballotta and Bonfiglioli (2016), while Dempster and Hong (2002) and Schneider and Tavin (2018) use multi-factor models based on stochastic volatility.

In this paper, we study a particular specification of the generalized bivariate normal distribution used by Fry-McKibbin et al. (2014) to analyze the impact of high-order co-moments on the risk associated with options written on contagious underlyings. The specification allows for co-volatility and co-skweness, and turns out to be particularly appropriate for the representation of non-linear dependence among asset returns by means of high-order co-moments. Under the specification considered in this paper, closed-form expressions of the conditional densities and the quasi-closed expressions for the marginal densities can be derived in a simple way. The relevant co-moments can also be computed by means of a one-dimensional numerical integral. This specification admits closed form formulae for single-name and exchange option in terms of one-dimensional integrals, which can be evaluated quickly. We also provide an approximated formula for spread option prices. The formula gives a lower bound for the true price. Nevertheless, extensive numerical experiments show that the bounds provided are very precise.

We use WTI versus Brent spread option prices to perform a day-to-day re-estimation of the joint (risk-neutral) probability distribution. Our dataset consists of European Call spread options with several maturities and strike prices, which allow the owner to buy WTI futures in exchange of Brent futures plus some extra cash, namely the strike price. The period under investigation covers a little more than a year of option prices including the months in which the effects of the worldwide pandemic named *Covid-19* have been dramatic for the energy sector. During those months, the futures on WTI have experienced a tremendous crash, due to the lack of oil demand and the limited storage facilities in the United States. In the same period, the Brent experienced a less pronounced but still strong negative impact. The use of this dataset is particularly valuable for our purposes for at least two reasons. First, an estimation exercise on a dataset which covers a period of crisis is useful in that it allows to test the quality of fit of the considered probability distribution in periods in which classic distributions usually fail. In other words, this dataset gives us the opportunity to test our specification during a period of stressed market conditions. Second, the dataset allows us to uncover the transmission channels of contagion in terms of risk-neutral high-order co-moments during that period. Then, we use the parameters obtained to highlight the contagion effects, in terms of high-order co-moments, that occurred in the last energy crisis due to the worldwide pandemic.

The remainder of the paper is organized as follows: in Sect. 2, we provide a specification of the generalized bivariate normal distribution which allows quasi-closed formulae for option prices. In Sect. 3, we apply the model to option pricing. The relevant formulae for the Greeks are also provided in Appendix B. In Sect. 4, we calibrate the model to WTI versus Brent spread option data. Section 5 assesses the impact of co-moments in risk management. Finally, in Sect. 6, we conclude.

## A tractable bivariate Generalized Normal model

We consider a world with two real-valued state variables denoted by $Y_1,Y_2$. Later in this paper we will interpret the state variables as the fundamental uncertainty driving returns of two asset in the context of option pricing and risk management. The standard assumption in option pricing is the normality of the fundamental uncertainty. The huge option pricing literature extends the normality assumption in many directions. In this paper we focus on the use of a particular specification of the generalized normal distribution considered by Fry et al. (2010) and Fry-McKibbin et al. (2014), who extend the work of Lye and Martin (1993) to the multidimensional case. In particular, we specify the density of the bivariate random variable $(Y_1,Y_2)$ as a special case of the generalized normal distribution proposed by Fry-McKibbin et al. (2014):

1 $$\begin{aligned} f_{Y_1,Y_2}(y_1,y_2)= \exp \left[ -\frac{1}{2}\theta _1 y_1^2 -\frac{1}{2}\theta _2 y_2^2 +\theta _3 y_1 y_2 +\theta _4 y_1 y_2^2 +\theta _5 y_1^2 y_2 -\theta _6 y_1^2 y_2^2 -\eta \right] \text {,} \end{aligned}$$

where $\theta _1=\theta _2 = \frac{1}{1-\rho ^2}$, $\theta _3 = \frac{\rho }{1-\rho ^2}$, $\rho \in (-1,1)$. We restrict our attention to the case $\theta _6 > 0$ to guarantee that the two random variables admit finite moments of all orders. Once the parameters $\theta _1,\ldots ,\theta _6$ are specified, $\eta$ is determined by the relation

$$\begin{aligned} \int \int _{\mathbb {R}^2} f_{Y_1,Y_2}(y_1,y_2)dy_1dy_2 = 1\text {.} \end{aligned}$$

From (1) we recover the bivariate normal density by setting $\theta _4=\theta _5=\theta _6 = 0$, where the parameters $\theta _1, \theta _2$ control the variance of $Y_1, Y_2$, respectively and $\theta _3$ drives the linear dependence between the states variables. The role of the remaining parameters is to allow for a more sophisticated dependence structure. In particular, $\theta _4$ and $\theta _5$ drive the co-skewness, that is the dependence of the bivariate random variables $(Y_1, Y_2^2)$ and $(Y_1^2,Y_2)$. In addition, $\theta _6$ models the interaction of the couple $(Y_1^2,Y_2^2)$, known as co-volatility.

**Fig. 1:**
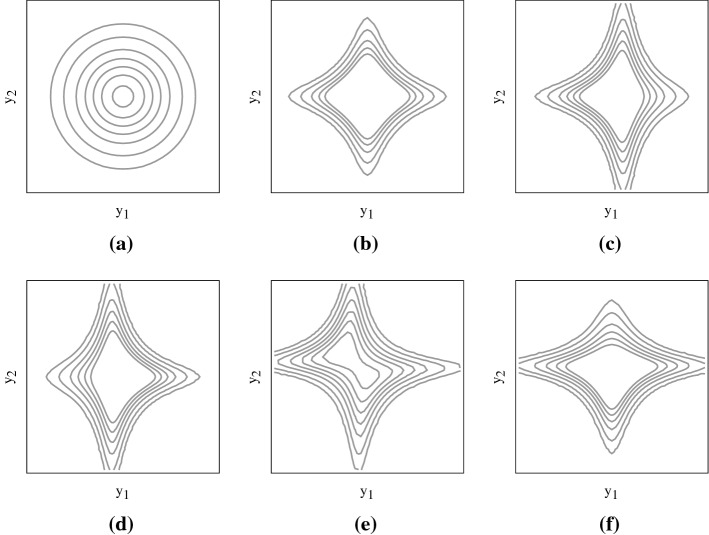
Contour plots of the density function (1) for different parametrization of the triple $\left( \theta _4,\theta _5,\theta _6\right)$. **a** (0, 0, 0); **b** (0, 0, 2); **c** (1.5, 0, 2); **d** $(-1.5,0,2)$; **e** (1.5, 1.5, 2); **f** (0, 1.5, 2); In all panels we set $\rho =0$

Figure 1 shows the effects of different values of these parameters. The parameters driving co-volatility distort symmetrically the density function, assigning more weight to events where one of the two components of the random variable is close to zero. The parameters driving co-skewness modify asymmetrically the previous effect, according to their sign and magnitude.

The probability density function in (1) allows for greater flexibility with respect to the normal distribution, while keeping analytic tractability. In fact, straightforward manipulation of the joint density function allows to derive conditional densities in closed form. Additionally, marginal densities can also be expressed in closed-form up to the solution of a one dimensional integral (which can be computed instantaneously) for the normalizing constant. These facts, which are summarized in the next proposition (see the proof in 1), turn out to be crucial for fast computation of option prices and model calibration. In terms of notation, here and throughout the rest of the paper $\phi (\cdot ;\mu ,\sigma ^2)$ denotes the density function of a normal random variable with expected value $\mu$ and variance $\sigma ^2$ whereas $\phi (\cdot )$ denotes the standard normal density function, i.e. $\phi (\cdot ;0,1)$. Finally $\varPhi (\cdot )$ denotes the cumulative distribution function of a standard normal random variable.

### Proposition 1

Consider the bivariate random variable $(Y_1,Y_2)$ with density given in (1). Suppose $\theta _5^2-2\theta _6\theta _1< 0$ and $\theta _4^2-2\theta _6\theta _2< 0$. Then:

1. $Y_1|Y_2=y_2$ is normally distributed, with expected value $\mu _{1|2}(y_2)$ and variance $\sigma ^2_{1|2}(y_2)$, where
  $$\begin{aligned} \sigma ^2_{1|2}(y_2)= \frac{1}{\theta _1+2\theta _6y_2^2-2\theta _5y_2}\text { and } \quad \mu _{1|2}(y_2)=(\theta _3y_2+\theta _4 y_2^2)\sigma ^2_{1|2}(y_2)\text {.} \end{aligned}$$
2. $Y_2|Y_1=y_1$ is normally distributed, with expected value $\mu _{2|1}(y_1)$ and variance $\sigma ^2_{2|1}(y_1)$, where
  $$\begin{aligned} \sigma ^2_{2|1}(y_1)=\frac{1}{\theta _2+2\theta _6 y_1^2-2\theta _4y_1} \text { and } \quad \mu _{2|1}(y_1)=(\theta _3y_1+\theta _5 y_1^2)\sigma ^2_{2|1}(y_1) \text {.} \end{aligned}$$
3. The marginal density of $Y_2$ is
  $$\begin{aligned} f_{Y_2}(y_2)= \frac{ 2\pi \textrm{e}^{-\eta }}{\sqrt{ \theta _2 }} \exp \left[ \frac{1}{2} (\theta _3y_2+\theta _4 y_2^2)^2 \sigma ^2_{1|2}(y_2) \right] \sqrt{ \sigma ^2_{1|2}(y_2)} \phi \left( y_2; 0, 1/\theta _2\right) \text {.} \end{aligned}$$
4. The marginal density of $Y_1$ is
  $$\begin{aligned} f_{Y_1}(y_1)= \frac{ 2\pi \textrm{e}^{-\eta }}{\sqrt{ \theta _1 }} \exp \left[ \frac{1}{2} (\theta _3 y_1+\theta _5 y_1^2)^2 \sigma ^2_{2|1}(y_1) \right] \sqrt{ \sigma ^2_{2|1}(y_1)} \phi \left( y_1; 0, 1/\theta _1\right) \text {.} \end{aligned}$$
5. The parameter $\eta$ can be computed by imposing that the integral over $\mathbb {R}$ of one of the two marginal densities above is unity, thus solving a one-dimensional integral.

Proposition 1 not only provides a representation of the density function in terms of a one-dimensional integral, but also suggests an algorithm for the simulation from such bivariate density.

1. Use the inverse CDF method to simulate a random variate, $y_2^*$, from the marginal density of $Y_2$, $f_{Y_2}(\cdot )$.
2. Use $y_2^*$ and the conditional density of $Y_1| Y_2$ and obtain $y_1^*$ by simulating from the normal distribution with mean $\mu _{1|2}(y_2^*)$ and variance $\sigma ^2_{1|2}(y_2^*)$.

Note that the inverse CDF needed in step 1. can be efficiently approximated by evaluating the non-normalized density at a properly selected grid of points (see Tsay, 2010, p.623). Therefore, the first step of the above algorithm only requires evaluating the kernel of $Y_2$ at a grid of points *without* having to compute the normalization constant $\eta$.

### Co-moments

In this section, we give formulae for the relevant co-moments (co-skewness and co-volatility). A first way to derive the formula for co-skewness (identified by looking at changes in the interaction between expected returns of the first asset and volatility of the second asset) is the following:

$$\begin{aligned} \mathbb {E}\left[ Y_1 Y_2^2 \right]= & {} \int _{\mathbb {R}} \int _{\mathbb {R}} y_1 y_2^2 f_{Y_1,Y_2}(y_1,y_2) \textrm{d}y_1 \textrm{d}y_2\\= & {} \int _{\mathbb {R}} y_1 \left[ \int _{\mathbb {R}} y_2^2 f_{Y_2|Y_1}(y_2|y_1)\textrm{d}y_2 \right] f_{Y_1}(y_1) \textrm{d}y_1 \\= & {} \int _{\mathbb {R}} y_1 \left\{ \left[ \mu _{2|1}(y_1)\right] ^2+\sigma ^2_{2|1}(y_1)\right\} f_{Y_1}(y_1) \textrm{d}y_1. \end{aligned}$$

Alternatively, the same measure of co-skewness can be obtained via

$$\begin{aligned} \mathbb {E}\left[ Y_1 Y_2^2 \right] = \int _{\mathbb {R}} y_2^2 \left[ \int _{\mathbb {R}} y_1 f_{Y_1|Y_2}(y_1|y_2)\textrm{d}y_1 \right] f_{Y_2}(y_2) \textrm{d}y_2 = \int _{\mathbb {R}} y_2^2 \mu _{1|2}(y_2) f_{Y_2}(y_2) \textrm{d}y_2 . \end{aligned}$$

Replicating the arguments above, it is straightforward to derive the formula for co-skewness identified by looking at changes in the interaction between expected returns of the second asset and volatility of the first asset:

$$\begin{aligned} \mathbb {E}\left[ Y_1^2 Y_2 \right] = \int _{\mathbb {R}} y_1^2 \mu _{2|1}(y_1) f_{Y_1}(y_1) \textrm{d}y_1 \end{aligned}$$

or

$$\begin{aligned} \mathbb {E}\left[ Y_1^2 Y_2 \right] = \int _{\mathbb {R}} y_2 \left\{ \left[ \mu _{1|2}(y_2)\right] ^2+\sigma ^2_{1|2}(y_2)\right\} f_{Y_2}(y_2) \textrm{d}y_2 \end{aligned}$$

Finally, co-volatility can be calculated as

$$\begin{aligned} \mathbb {E}\left[ Y_1^2 Y_2^2 \right] = \int _{\mathbb {R}} y_2^2 \left\{ \left[ \mu _{1|2}(y_2)\right] ^2+\sigma ^2_{1|2}(y_2)\right\} f_{Y_2}(y_2) \textrm{d}y_2 \end{aligned}$$

or as

$$\begin{aligned} \mathbb {E}\left[ Y_1^2 Y_2^2 \right] = \int _{\mathbb {R}} y_1^2 \left\{ \left[ \mu _{2|1}(y_1)\right] ^2+\sigma ^2_{2|1}(y_1)\right\} f_{Y_1}(y_1) \textrm{d}y_1. \end{aligned}$$

Note that the formula for co-moments can also be derived using the results derived by Fry et al. (2010). To recall their result, let us write the log of the bivariate density as

$$\begin{aligned} \ell =\log f_{Y_1,Y_2}(y_1,y_2)=h(y_1,y_2)-\eta , \end{aligned}$$

with

$$\begin{aligned} \eta =\log \left[ \int \int _{\mathbb {R}^2} \textrm{e}^{h(y_1,y_2)}\textrm{d}y_1 \textrm{d}y_2\right] \end{aligned}$$

and let us denote by $\theta$ the vector of parameters. Then

$$\begin{aligned} \frac{\partial \ell }{\partial \theta } = \frac{\partial h}{\partial \theta } - \frac{\partial \eta }{\partial \theta }. \end{aligned}$$

Fry et al. (2010) proved that

2 $$\begin{aligned} \frac{\partial \eta }{\partial \theta } = \mathbb {E}\left[ \frac{\partial }{\partial \theta }h(Y_1,Y_2)\right] . \end{aligned}$$

Our representation of the relevant co-moments is not as elegant as that provided in (2). Nevertheless, it provides a practical and quick way to compute the relevant quantities. The representation of Fry et al. (2010) is indeed in implicit form, since the expression for $\eta$ is not available.

## Generalized normal distribution and option prices

In this section, we propose a lower bound for European spread options which pays at the maturity $\tau$ the amount $c_\tau = (S_{1,\tau } -S_{2,\tau } - K)^+$, where $S_{1,\tau }, S_{2,\tau }$ are the value at maturity of the two underlying assets, *K* is the strike price and $x^+$ denotes the positive part of *x*. In the special case $K=0$, the lower bound turns out to be exact, thus generalizing Margrabe (1978)’s formula for European exchange options. In the case $K > 0$, the lower bound is very close to the real price, thus providing a fast and reliable way to compute spread option prices under our specification for the generalized normal distribution.

We specify the risk-neutral probability distribution of the underlying assets along the lines of Fry-McKibbin et al. (2014). We consider a financial market with two risky assets and denote by *r* the instantaneous risk-free rate. Our standing assumption is about the distribution of the two underlying assets at maturity $\tau$, which we specify directly under the risk-neutral probability measure:

3 $$\begin{aligned} S_{i,\tau }=S_{i,0}\textrm{e}^{({\bar{r}} - \frac{\sigma _i^2}{2} {-\lambda _i} )\tau + \sigma _i\sqrt{\tau }Y_i} \text {.} \end{aligned}$$

for $i=1,2$. In (3), the distribution of the random vector $(Y_1,Y_2)$ is described by (1), the parameters $\sigma _i$ is the volatility of asset *i*,

$$\begin{aligned} {\bar{r}} = {\left\{ \begin{array}{ll} r &{} \text { if the underlying assets are stocks} \\ 0 &{} \text { if the underlying assets are futures} \end{array}\right. } \end{aligned}$$

and $\lambda _i= \frac{1}{\tau } \log m_{Y_i}(\sigma _i\sqrt{\tau }) - \frac{\sigma _i^2}{2}$, where $m_{Y_i}(\cdot )$ is the moment generating function of $Y_i$.

The value of a European spread option^2^ written on the two assets defined in (3) is given by

$$\begin{aligned} c_t =\textrm{e}^{-r(\tau -t)} \mathbb {E}_t\left[ \left( S_{1,\tau }-S_{2,\tau } - K\right) ^+\right] . \end{aligned}$$

Apart from the special case $K=0$, no closed form solutions exist for such contracts. However, the literature offers a fast way to compute spread option prices by means of convenient approximations. In this context, Kirk (1995) proposes a first approximation, subsequently refined by Bjerksund and Stensland (2014). Caldana and Fusai (2013) generalize Bjerksund and Stensland (2014) to any model where the joint characteristic function can be computed explicitly. The approximation proposed in Bjerksund and Stensland (2014) produces option prices which are very close to the true price. It consists in substituting the payoff at maturity with the following approximated payoff:

4 $$\begin{aligned} {\tilde{c}}_{\tau } = \left( S_{1,\tau } - S_{2,\tau } -K\right) I\left( S_{1,\tau }\ge \frac{a }{c}S_{2,\tau }^b\right) , \end{aligned}$$

where

$$\begin{aligned} a=S_{2,t}\textrm{e}^{{\bar{r}}(\tau -t)} +K, \qquad b=\frac{S_{2,t}\textrm{e}^{{\bar{r}}(\tau -t)}}{a}, \qquad c=\mathbb {E}_t\left[ S_{2,\tau }^b\right] . \end{aligned}$$

With our distributional assumptions, this leads to option prices that are very rough (sometimes too rough) approximations of the true price. However, a slight modification of the approximation above, which consists in setting $c = S_{2,t} \textrm{e}^{{\bar{r}}(\tau -t)}$ instead of $c = \mathbb {E}_t\left[ S_{2,\tau }^b\right]$, proves to be effective. In the next proposition, we compute the approximated option prices.

### Proposition 2

Under the assumptions of Proposition 1, the quantity

$$\begin{aligned} {\tilde{c}}_t = \textrm{e}^{-r(\tau -t)}\mathbb {E}_t\left[ {\tilde{c}}_\tau \right] \end{aligned}$$

reduces to

5 $$\begin{aligned} \begin{aligned} {\tilde{c}}_t&= \textrm{e}^{-r(\tau -t)} \int _{\mathbb {R}} \left\{ \left[ {S_{1,\tau }\left( y_1\right) } -K \right] \varPhi \left[ {\tilde{d}}_1(y_1) \right] \right. \\&\quad \left. -\,S_{2,t} \exp \left[ c_2 + \alpha _2\mu _{2|1}(y_1) +\frac{\alpha _2^2}{2}\sigma ^2_{2|1}(y_1) \right] \varPhi \left[ {\tilde{d}}_2(y_1) \right] \right\} f_{Y_1}(y_1) \textrm{d}y_1\text {,} \end{aligned} \end{aligned}$$

where

$$\begin{aligned} {\tilde{d}}_1(y_1)= \frac{\frac{\sigma _1 y_1}{b \sigma _2}-{\tilde{h}}-\mu _{2|1}(y_1)}{\sqrt{\sigma ^2_{2|1}(y_1)}}; \qquad {\tilde{d}}_2(y_1)= {\tilde{d}}_1(y_1) - \alpha _2\sqrt{\sigma ^2_{2|1}(y_1)} \text {,} \end{aligned}$$

and

$$\begin{aligned} {\tilde{h}}= & {} \frac{\log \left( \frac{a {S_{2,t}^b} }{c S_{1,t}}\right) -\left( {\bar{r}}(1-b) + \frac{\sigma _2^2{b} -\sigma _1^2}{2} + {\lambda _2 b - \lambda _1} \right) (\tau -t)}{b \sigma _2 \sqrt{\tau -t}};\\{} & {} \quad c_2 = \left( {{\bar{r}}} - \frac{\sigma _2^2}{2} -{\lambda _2} \right) {(\tau -t)}; \quad \alpha _2 = \sigma _2\sqrt{\tau -t}\text {.}\end{aligned}$$

We observe that (5) can be considered as an approximation for spread option prices only for the case $K\ge 0$. The complementary case can be easily dealt with, because the call option can be thought of a put on the opposite price spread, i.e. $S_{2,\tau } - S_{1,\tau }$, and put values are obtained using the put-call parity (see the discussion at the beginning of Sect. 4).

Hedging the risk associated with trading in financial options involves the use of the so-called Greeks. These are the derivatives (in the mathematical sense) of the option price with respect to some quantities of interest. Two widely used hedging strategies for spread options are delta hedging and delta-gamma hedging (see, for instance, Venkatramanan and Alexander, 2011). The former involves taking positions on the underlying stocks, proportionally to the derivative of the option price with respect to each underlying, the so-called deltas of the option. The latter implies having in the hedging portfolio not only some shares of the stocks, but also some options on the two stocks, in a way proportional to the gammas of the option (the second derivatives of the option prices with respect to the underlying stocks). Both hedging strategies require the frequent rebalancing of the hedging portfolio, calling for a fast way to compute the necessary hedge ratios. In Appendix B, we provide the deltas and the gammas for the option price formula (5).

The formula in (5), which is exact in the special case of European exchange options, reduces the two-dimensional option pricing problem to the solution of one-dimensional integral, which can be easily and quickly computed with commonly available routines. This makes fast calibration of spread options possible. Although we are not the first who are able to calibrate such options (see, for instance, Schneider and Tavin, 2018) the approximation provided in (5) allows, for the first time, the use of high order co-moments in the calibration. However, before proceeding with the applications of the models, we perform a series of numerical experiments to evaluate the quality of the approximation provided by (5).

### Quality of the approximation

To show the quality of the approximation detailed in Proposition 2, we run a set of numerical evaluations by comparing the approximated option prices given in (5) with the *true* prices. We refer to the true option price as the one computed by a routine for two-dimensional numerical integration, using the original representation for the density given in formula (1).

We consider WTI versus Brent hypothetical spread options allowing the owner to exchange a futures on the WTI with a futures written on Brent at a given maturity. We set $S_{1,t} = 51.26$ and $S_{2,t} = 55.4$. These are the New York Mercantile Exchange (NYMEX) closing quotes, as observed on March 20, 2020, of the futures written on WTI and Brent, respectively with delivery on September 1, 2020. At the same date, we observe several spread options written on the two underlying futures, and several strike prices, ranging from $K = 0$ to $K = 7$. We use these parameters, in conjunction with a risk-free interest rate $r = 0.007$ and $\sigma _1=0.25$, $\sigma _2 = 0.2$. To isolate the effects of parameters governing co-movements on the approximation quality, we consider 6 different sets of parameters’ values. In particular, we fix the set $\varLambda = \{-0.9,-0.5,-0.1,0.1,0.5,0.9\}$ and define:

- $$\varTheta _1{:}{=} \varLambda \times \varGamma _1 \times \varGamma _1 \times \{0.25\}$$ with $$\varGamma _1 {:}{=} \{-0.4, -0.3, -0.2,-0.1,0, 0.1,0.2,0.3,0.4\}$$;
- $$\varTheta _2{:}{=} \varLambda \times \varGamma _2 \times \varGamma _2 \times \{0.5\}$$ with $$\varGamma _2 {:}{=} \{-0.7, -0.5, -0.3,-0.1, 0.1,0.3,0.4\}$$;
- $$\varTheta _3{:}{=} \varLambda \times \varGamma _3 \times \varGamma _3 \times \{1\}$$ with $$\varGamma _3 {:}{=} \{-0.9, -0.7, -0.5,-0.3,-0.1, 0.1,0.3,0.5,0.7,0.9\}$$;
- $$\varTheta _4{:}{=} \varLambda \times \varGamma _4 \times \varGamma _4 \times \{2\}$$ with $$\varGamma _4 {:}{=} \{-1.4, -1, -0.6,-0.2, 0.2,0.4,0.6,1,1.4\}$$;
- $$\varTheta _5{:}{=} \varLambda \times \varGamma _5 \times \varGamma _5 \times \{4\}$$ with $$\varGamma _5 {:}{=} \{-1.9, -1.5, -1.1,-0.7,-0.3, 0.3,0.7,1.1,1.5,1.9\}$$;
- $$\varTheta _6{:}{=} \varLambda \times \varGamma _6 \times \varGamma _6 \times \{8\}$$ with $$\varGamma _6 {:}{=} \{-2.8, -2.4, -2,-1.6,-1.2, -0.8,-0.4,0.4,0.8,1.2,1.6,2,2.4,2.8\}$$;

The values in the sets $\varTheta _1$–$\varTheta _6$ are chosen to span the relevant parameter space as much as possible. In practice, we selected $\rho$ between − 0.9 and 0.9, $\theta _4,\theta _5$ between -2.8 and 2.8 and $\theta _6$ between 0.25 and 8. The choice of this ranges for the parameters related to the co-movements has been made based on the estimation results presented in the next section. In fact, for our day-to-day re-estimation we fitted values of $\theta _4,\theta _5$ ranging roughly from − 2 to 2 and fitted values of $\theta _6$ ranging rougly from 0.3 to 6.

**Table 1:** For column $\varTheta _i$, each element of the table is defined as ${\max }_{(\rho ,\theta _4, \theta _5, \theta _6) \in \varTheta _i} \frac{c_t - {\tilde{c}}_t}{c_t}$ where ${\tilde{c}}_t$ is the lower bound derived in proposition 2, and $c_t$ is the true price obtained by means of a numerical routine for two-dimensional integration

Strike	$\varTheta _1$	$\varTheta _2$	$\varTheta _3$	$\varTheta _4$	$\varTheta _5$	$\varTheta _6$
0	3.91E−05	6.12E−05	5.93E−07	7.41E−05	3.31E−05	2.49E−05
0.5	3.87E−03	3.74E−03	4.43E−03	9.82E−03	9.38E−04	2.54E−03
1	2.48E−02	4.88E−02	8.39E−02	8.18E−03	1.27E−02	7.52E−03
2	6.01E−02	8.82E−02	5.24E−02	7.04E−02	9.79E−02	6.86E−02
5	1.82E−01	3.97E−01	3.06E−01	2.47E−01	1.40E−01	9.81E−02
6	6.58E−01	5.98E−01	5.09E−02	5.25E−01	4.01E−01	5.36E−01
7	5.22E−01	4.32E−01	4.02E−01	3.85E−02	6.37E−01	4.48E−01

The errors are expressed in percentage

We compare the true option prices, obtained by means of two-dimensional quadrature, with the lower bounds obtained by applying a one-dimensional quadrature to (5). We present the results in Table 1. The approximation provided by the lower bound deteriorates as the option goes deep out of the money. This effect is clearly expected and often encountered in such kinds of approximation [see, for instance, the extensive numerical experiments in Caldana and Fusai (2013)]. Nevertheless, the approximation quality is satisfactory and unaffected by the parameters governing co-movements.

## Applications to option pricing

The approximation provided in Proposition 2 allows for fast estimation of the risk-neutral joint probability distribution. In this section, we evaluate the quality of fit when estimation is performed on observed spread option prices. We also provide empirical evidence of the fact that the fundamental uncertainty implied from spread option prices can be far from being normally distributed.

**Table 2:** Number of option prices available, classified for different levels of moneyness and maturities.

	Days to maturity				
Moneyness	90–180	180–360	360–540	> 540	All
1.05–1.15	173	1308	640	8	2129
1.15–1.25	340	1511	1616	117	3584
1.25–1.35	160	250	27	31	468
${>}\,1.35$	29	31	0	0	60
All	702	3100	2283	156	6241

Moneyness is measured as $\frac{|K|+S^{Brent}_0}{S^{WTI}_0}$

**Fig. 2:**
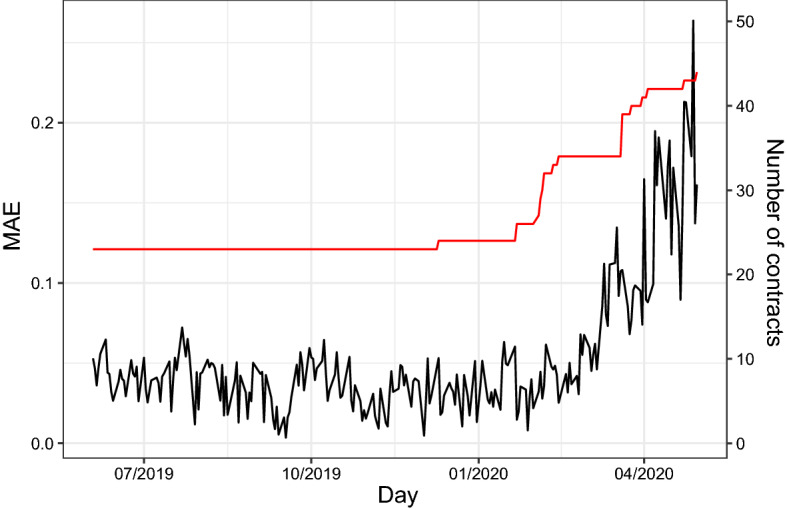
Estimation error, measured in terms of mean absolute error, for each of the 231 trading dates in which we have performed the calibration. The red line indicates the number of observation available in each calibration day. Its scale is indicated in the right side of the *y*-dimension of the plot

Our dataset consists of WTI-Brent spread option closing prices along with WTI and Brent future quotes at NYMEX, downloaded from Bloomberg. This option pays at maturity *T* the amount $\left( S^{WTI}_T - S^{Brent}_T - K\right) ^+$. Given that in our dataset all stike prices are negative, we exploit the put-call-parity for European options in the following way. Each available European call option on the spread $S^{WTI}_T - S^{Brent}_T$ and negative strike price *K* is actually an option giving the right to sell at maturity the spread $S^{Brent}_T - S^{WTI}_T$ at the strike price |*K*|, i.e. a European Put option with payoff $\left( |K| + S^{Brent}_T - S^{WTI}_T\right) ^+$. However, given that we only have an approximation formula for call options, we first apply formula (5) to compute the price of the corresponding European call option, with payoff $\left( S^{WTI}_T - S^{Brent}_T - |K|\right) ^+$, and then use the put-call-parity to recover the European put option price. Further, we measure the levels of options moneyness as $\frac{|K|+S^{Brent}_0}{S^{WTI}_0}$, being $S^{WTI}_0, S^{Brent}_0$ the initial levels of the underling futures. In Table 2, we report the number of options available for different levels of moneyness and maturities. The time period spans from June 3, 2019, to April 30, 2020, for a total of 231 trading dates. For each trading date, we have a set of prices of spread options with different strike prices and maturities and the corresponding value of the underlying futures. We estimate the probability distribution for each of the 231 dates available, by selecting the parameters $\sigma _1,\sigma _2,\rho , \theta _4,\theta _5,\theta _6$ that minimize the sum of squared difference between the observed option prices and the theoretical option prices from our specification. Then, we evaluate the quality of fit of the model by both in terms of Mean Absolute Error (MAE), that is the averaged absolute value of the difference between observed and theoretical option prices and in terms of Mean Relative Error (MRE), defined as the averaged absolute value of the relative error. In Fig. 2, we plot the calibration error in terms of MAE for each of the 231 days of calibrated option prices. The figure clearly shows the ability of the considered probability distribution to replicate observed option prices. The deterioration of the quality of fit observed in the last part of the plot reflects the turbulence observed in the oil market due to the start of the Covid-19 pandemic in february 2020. In that period, the futures on WTI experienced a dramatic crash due to the lack of storage facilities. Despite this fact, the loss in quality of fit seems to be quite moderate if compared with the tremendous turmoil observed in the market during those months. This bring us to the conclusion that our specification is able to reproduce observed option prices even in periods of unprecedented turbulence. In Table 3, we report the MREs, classified by maturities and levels of moneyness. The table reveals a satisfactory fit in terms of relative error. Higher levels of moneyness seem to deteriorate the goodness of fit of the model, although the deterioration seems to be restrained. The impact of different levels of maturity on the quality of the fit appears to be random, as no clear patterns are identifiable. Overall, our analysis leads us to conclude in favor of the particular specification of the generalized bivariate normal distribution proposed in this paper, in terms of its ability to replicate observed option prices, even in presence of high turmoil in the market.

**Table 3:** Mean Relative Errors of the estimation, classified by different levels of moneyness and maturities

Moneyness	Days to maturity				
	90–180	180–360	360–540	> 540	All
1.05–1.15	3.39	3.05	3.14	2.95	3.08
1.15–1.25	4.75	4.91	5.12	5.08	4.87
1.25–1.35	4.82	4.61	4.99	5.10	4.77
${>}\,1.35$	5.43	5.06	–	–	5.32
All	3.89	4.25	4.15	4.05	4.49

Moneyness is measured as $\frac{|K|+S^{Brent}_0}{S^{WTI}_0}$. The MREs are expressed in percentages

**Fig. 3:**
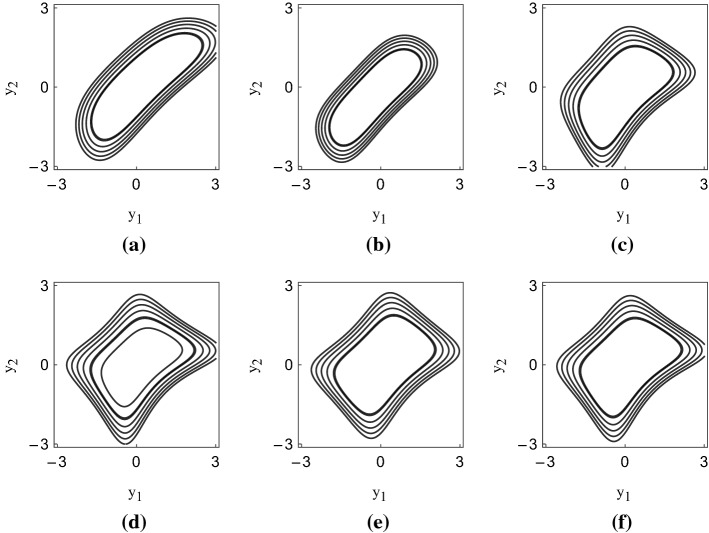
Estimated risk-neutral density functions, first set. The figure shows the contours of the implicit density functions of the couple $(Y_1,Y_2)$ for different calibration days. **a** refers to 2019-06-03; **b** 2019-07-01; **c** 2019-08-27; **d** 2019-09-25; **e** 2019-11-06; **f** 2019-11-20

To illustrate how the WTI-Brent’s co-movements are affected by high-order co-moments, we select 12 trading dates across the period under investigation and show the implied density functions of the couple $(Y_1,Y_2)$ estimated during those dates. The couple drives the fundamental uncertainty in the market.^3^ In Fig. 3, we plot the first set of estimated density functions. These plots refer to the period of time between September and November, 2019. Despite the fact that during those months the oil market was experiencing a time of relative calm, the density functions implied by our specification of the generalized bivariate normal distribution with high-order co-moments are far from following a bivariate normal distribution. In particular, all six panels show signs of abundant (risk neutral) co-skewness. Co-volatility is also abundant, especially in the last four panels of Fig. 3.

**Fig. 4:**
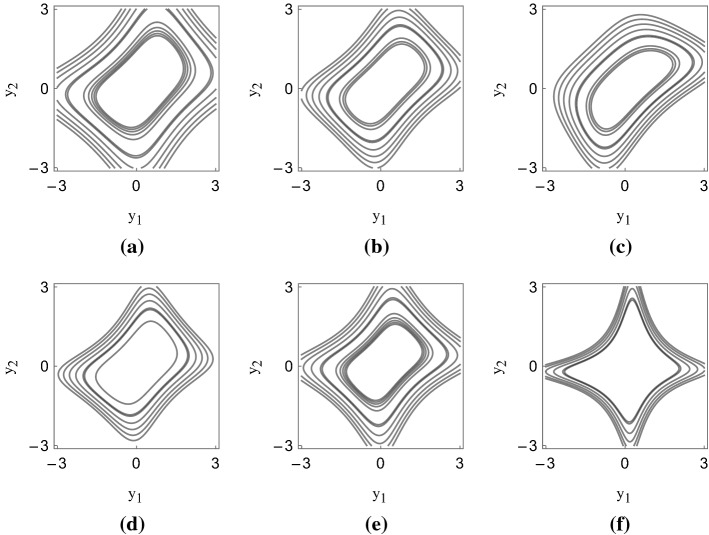
Calibrated density functions, second set. The figure shows the contours of the implicit density functions of the couple $(Y_1,Y_2)$ for different calibration days. **a** refers to 2019-12-05; **b** 2020-01-21; **c** 2020-02-4; **d** 2020-03-04; **e** 2020-04-16; **f** 2020-04-30

In Fig. 4, we plot the second set of implied density functions. The second row of the figure is particularly interesting, since it refers to the months in which the oil market was experiencing the tremendous crash provoked by the fall of the demand subsequent to the world-wide pandemic. The implied density functions show patterns in which co-skewness and co-volatility are prominent, showing that high-order (risk-neutral) co-moments are important transmission channels of contagion in the WTI-Brent market during the last turbulent period.

## Generalized normal distribution and risk management

In this section we provide a further applications aimed at evaluating the impact of co-movements on risk management, in terms of risk measures like Value-at-Risk (VaR) and Expected Shortfall (ES).

**Fig. 5:**
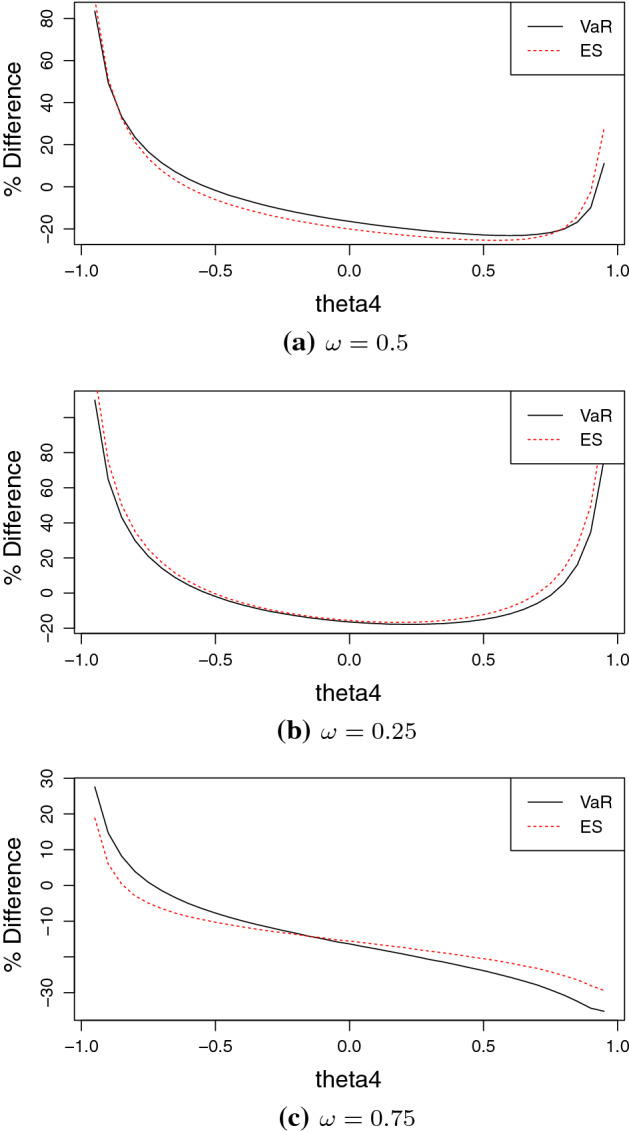
Relative percentage differences $\left( VaR^1_{5\%}(R_p)/VaR^N_{5\%}(R_p)-1 \right) \times 100$ (red lines) and $\left( ES^1_{5\%}(R_p)/ES^N_{5\%}(R_p)-1 \right) \times 100$ (black lines) between risk measures for the portfolio return (7) based on log-returns from equation (6) and those from a bivariate normal distribution

In what follows, we consider two stocks $S_{1},S_{2}$, whose future behavior after $\tau$ days is modeled, under the historical probability measure, by:

6 $$\begin{aligned} S_{i,\tau } = S_{i,0} e^{\mu _i \tau + \sigma _i \sqrt{\tau } Y_i}\text {,} \end{aligned}$$

where the density of the bivariate random variable $(Y_1,Y_2)$ is given in (1).^4^ We set the time horizon to $\tau =1$, denote by $X_1,X_2$ the logarithmic returns of the two stocks, and focus on risk measures for the portfolio with return described by the random variable

7 $$\begin{aligned} R_p=\omega X_1+(1-\omega ) X_2 \end{aligned}$$

where $\omega \in [0,1]$ is the weight of the first asset. The main goal of the section is to assess whether the presence of co-movements increases the two risk measures compared to the normal case. In particular, in both cases, we compute for the portfolio the 5%-VaR and 5%-ES and report the relative percentage differences $\left( VaR^1_{5\%}(R_p)/VaR^N_{5\%}(R_p)-1 \right) \times 100$ and $\left( ES^1_{5\%}(R_p)/ES^N_{5\%}(R_p)-1 \right) \times 100$, where the risk measures with superscript ‘1’ and ‘N’ are the ones from model (1) and the normal model, respectively. We investigate the effect of co-skewness on VaR/ES and therefore make the $\theta _4$ parameter vary in the interval $(-0.95,0.95)$ and fix the remaining parameters as follows: $\rho =\theta _5=0$, and $\theta _6=0.5$. For the normal distribution we assume zero correlation and hence this case is also obtained from (1) setting $\rho =\theta _5=\theta _5=\theta _6=0$. In addition, we set $\mu _1=\mu _2 = 0$ and $\sigma _1=\sigma _2=1$. This allows us to isolate the effects of co-movements, in line with the objectives of our paper. We consider an equally-weighted portfolio ($\omega =0.5$), one with a weight $\omega =0.25$ and one with $\omega =0.75$.^5^ Results, reported on Fig. 5, are obtained using the Monte Carlo method to estimate the two risk measures, based on the average of 10,000 estimates (each obtained from a sample of length 1,000). For both $\omega =0.5$ and $\omega =0.25$ the graphs are u-shaped. In particular, when $\omega =0.25$, the two risk measures are severely underestimated by the normal model when the value of the parameter associated to co-skewness is larger (in absolute value) than one half. Furthermore, in Panel (b) the line corresponding to ES lies consistently above the one for VaR, meaning that in this case the normal model underestimates ES more severely, at least for values of $\theta _4$ near plus or minus one. Interestingly, when $\omega =0.75$, the relative percentage differences are decreasing with $\theta _4$. The two risk measures calculated for this portfolio are larger for the model allowing for co-movements only for values of $\theta _4$ very close to minus one.

We also make a comparison in terms of the following conditional Expected Shortfall

$$\begin{aligned} CES_{\alpha _1,\alpha _2}(X_1,X_2)=-\mathbb {E}[X_1|X_1<-VaR_{\alpha _1}(X_1),X_2<-VaR_{\alpha _2}(X_2)] \\ = -\frac{\int _{-\infty }^{-VaR_{\alpha _1}(X_1)} \int _{-\infty }^{-VaR_{\alpha _2}(X_2)} x_1 f_{Y_1,Y_2}\left( \frac{x_1-\mu _1}{\sigma _1},\frac{x_2-\mu _2}{\sigma _2}\right) \textrm{d}x_1 \textrm{d}x_2}{ \int _{-\infty }^{-VaR_{\alpha _1}(X_1)} \int _{-\infty }^{-VaR_{\alpha _2}(X_2)} f_{Y_1,Y_2}\left( \frac{x_1-\mu _1}{\sigma _1},\frac{x_2-\mu _2}{\sigma _2}\right) \textrm{d}x_1 \textrm{d}x_2 } . \end{aligned}$$

The above quantity has been recently investigated in the context of the bivariate Kumaraswamy distribution by Ghosh and Marques (2021) and is particularly useful to measure the overflow of the two risks $X_1$ and $X_2$ at the same time.

**Fig. 6:**
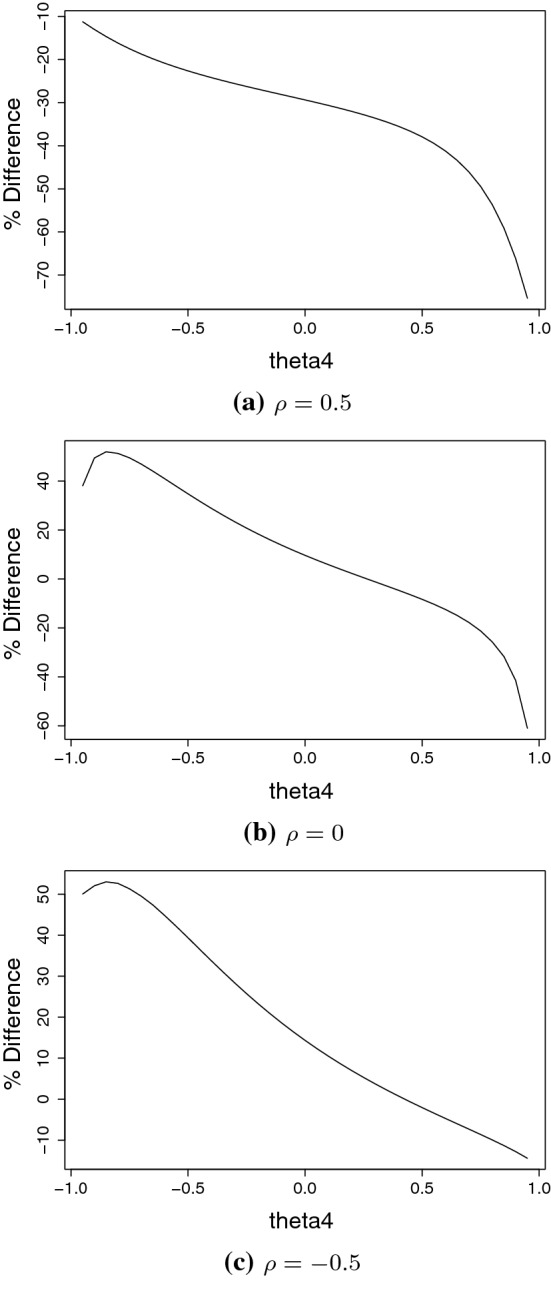
Relative percentage differences $\big (CES^1_{5\%,5\%}(X_1,X_2)/CES^N_{5\%,5\%}(X_1,X_2)\big )\times 100$ between conditional ES based the log-returns from Eq. (6) and those from a bivariate normal distribution

We calculate relative percentage differences between model (1) and the normal one. For the former we make the $\theta _4$ parameter vary in the interval $(-0.95,0.95)$ and set $\theta _5=0.75$ and $\theta _6=0.5$. Also, to isolate the effect of co-movements, again we set $\mu _1=\mu _2 = 0$ and $\sigma _1=\sigma _2 = 1$. Relative percentage differences are given in Fig. 6. For both models, we consider three different values for the $\rho$ parameters, i.e. $\rho \in \{-0.5,0,0.5\}$. It is clear that for $\rho =0.5$ the conditional Expected Shortfalls from the normal model are always larger than the measures based on the bivariate density (1). However, for $\rho =0$ and $\theta _4<0$ and for $\rho =-0.5$ and $\theta _4<0.5$ we observe that the considered risk measure is larger for model (1).

**Fig. 7:**
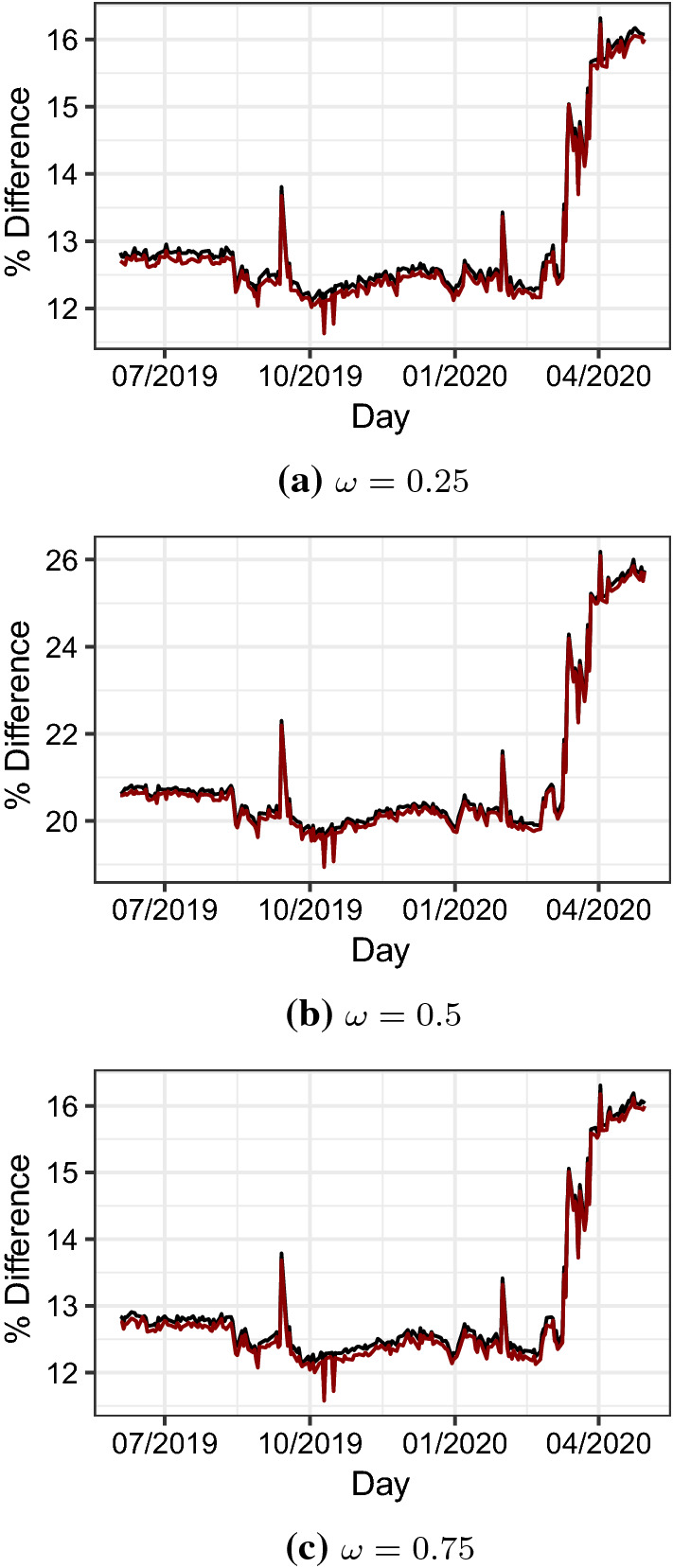
Relative percentage differences $\left( VaR^1_{5\%}(R_p)/VaR^N_{5\%}(R_p)-1 \right) \times 100$ (red lines) and $\left( ES^1_{5\%}(R_p)/ES^N_{5\%}(R_p)-1 \right) \times 100$ (black lines) between risk measures for the portfolio return $R_p=\omega X_1+(1-\omega ) X_2$. The risk factors $(X_1,X_2)$ denote log-returns from SPX and XAU, respectively and are modelled either as the one-day-ahead log-returns from Eq. (6) or via the bivariate normal distribution

To complete this section, we use real data to evaluate the impact of co-movements on risk measures like VaR and ES. The aim is to compare risk measures for the portfolio return (7) and $(X_1,X_2)$ described by density (1) and those based on a normal model. We consider a pair of assets, namely the S &P 500 index (Ticker SPX) and gold (Ticker XAU). The rationale for selecting these two assets is that, contrary to the previous case, they allow diversification in a portfolio. We download 1,231 daily observations for log-returns of the two assets and assume that $(X_1,X_2)$ are the log-returns from SPX and XAU, respectively and are modelled either as the one-day-ahead log-returns from Eq. (6) or via the bivariate normal distribution. We estimate the parameters of the model with the maximum likelihood (ML) method using daily data and a rolling window of 1000 observations. The set of estimated parameters covers the same period of the previous analysis, i.e. June 3, 2019 to April 30, 2020. We use the estimated parameters to derive portfolio VaR and ES and do the same in the normal case. In Figure 7 we plot the time series of relative percentage differences between risk measures calculated based on (1) and based on a bivariate normal distribution. A few remarks are in order. Firstly, for all the days in the sample and for all the values of $\omega$ we consider, risk measures that take into account co-movements are larger than the normal-based measures. Indeed, VaR and ES based on (1) and on the estimated parameters are about 11% to 26% larger that the same measures derived based on the bivariate normal model. Secondly, the relative percentage differences are numerically close in the case of a 25/75 or 75/25 portfolio and larger in the case of the 50/50 portfolio of S &P 500 and gold. Lastly, we observe a substantial increase in the series of relative percentage differences during the months of the pandemic.

## Conclusions

In this paper, we studied a specification of the generalized bivariate normal distribution which combines great flexibility with simple and fast computations for option pricing. The considered probability distribution allows to take into accounts high-order co-moments, namely co-skewness and co-volatility, when modeling financial uncertainty. It is a special case of Fry-McKibbin et al. (2014), but has the important advantage of being tractable. Indeed, by conditioning arguments, most important quantities of interest can be expressed in terms of one dimensional integral, which in turn can be evaluated quickly.

We derive a closed form formula for exchange option prices, thus generalizing Margrabe (1978), and an approximated formula for spread option prices and greeks which turns out to be very accurate. We exploited the gain in speed [with respect to the more general model of Fry-McKibbin et al. (2014)], due to the tractability of the density function, to perform 231 calibration exercises, one for each trading dates in the available dataset, on WTI-Brent spread options traded at NYMEX. Our exercise shows that: *i*) The probability distribution provides a good fit for spread option data also in turbulent periods and *ii*) The implied density functions display patterns in which co-volatility and co-skewness play an important role.

Finally, we showed some risk management applications of the proposed setup. Based also on real data on the S &P 500 index and gold we highlighted the effects of high-order co-moments in the measurement of VaR and ES. We do believe that a further investigation of generalized bivariate normal distribution in the lines of making high-order co-moments dynamic would be of great practical interest.

## A Proofs

### A.1 Proof of Proposition 1

To derive the conditional density of $Y_1$ given $Y_2$, it suffices to write the logarithm of (1) as

$$\begin{aligned}{} & {} \log f_{Y_1,Y_2}(y_1,y_2)= -\frac{1}{2}\theta _2 y_2^2 -\frac{1}{2}\left[ (\theta _1+2\theta _6y_2^2-2\theta _5y_2) y_1^2 -2(\theta _3y_2+\theta _4y_2^2) y_1 \right] -\eta \\{} & {} \quad =-\frac{1}{2}\theta _2 y_2^2 -\frac{1}{2}(\theta _1+2\theta _6y_2^2-2\theta _5y_2)\left[ y_1^2 -2\frac{\theta _3y_2+\theta _4 y_2^2}{\theta _1+2\theta _6y_2^2-2\theta _5y_2}y_1 \right] -\eta \end{aligned}$$

Using the notation $\sigma ^2_{1|2}(y_2)= \frac{1}{\theta _1+2\theta _6y_2^2-2\theta _5y_2}$, $\mu _{1|2}(y_2)=\frac{\theta _3y_2+\theta _4 y_2^2}{\theta _1+2\theta _6y_2^2-2\theta _5y_2} =(\theta _3y_2+\theta _4 y_2^2)\sigma ^2_{1|2}(y_2)$, and completing the square gives

$$\begin{aligned} \log f_{Y_1,Y_2}(y_1,y_2)= & {} -\frac{1}{2}\theta _2 y_2^2+ \frac{1}{2} (\theta _3y_2+\theta _4 y_2^2)^2\sigma ^2_{1|2}(y_2)\\{} & {} -\frac{1}{2\sigma ^2_{1|2}(y_2)}\left[ y_1 - \mu _{1|2}(y_2) \right] ^2-\eta \end{aligned}$$

Therefore, the joint density can be written as

8 $$\begin{aligned} f_{Y_1,Y_2}(y_1,y_2)= & {} \frac{ 2\pi \textrm{e}^{-\eta }}{\sqrt{ \theta _2 }} \exp \left[ \frac{1}{2} (\theta _3y_2+\theta _4 y_2^2)^2 \sigma ^2_{1|2}(y_2) \right] \sqrt{ \sigma ^2_{1|2}(y_2)} \phi \left( y_2; 0, 1/\theta _2\right) \nonumber \\{} & {} \quad \phi \left( y_1; \mu _{1|2}(y_2), \sigma ^2_{1|2}(y_2) \right) . \end{aligned}$$

From (8) it immediately follows that

9 $$\begin{aligned} Y_1|Y_2=y_2\sim N\left( \mu _{1|2}(y_2), \sigma ^2_{1|2}(y_2)\right) \end{aligned}$$

Note that for the conditional variance of $Y_1|Y_2=y_2$ to be positive, it is required that $\theta _5^2-2\theta _6\theta _1< 0$. Furthermore, integrating (8) with respect to $y_1$, the marginal density of $Y_2$ is obtained as

10 $$\begin{aligned} f_{Y_2}(y_2)= \frac{ 2\pi \textrm{e}^{-\eta }}{\sqrt{ \theta _2 }} \exp \left[ \frac{1}{2} (\theta _3y_2+\theta _4 y_2^2)^2 \sigma ^2_{1|2}(y_2) \right] \sqrt{ \sigma ^2_{1|2}(y_2)} \phi \left( y_2; 0, 1/\theta _2\right) . \end{aligned}$$

By imposing that the integral of $$ f_{Y_2}(y_2)$$ over $$\mathbb {R}$$ is unity, one finds that

$$\begin{aligned} \textrm{e}^{\eta }= \frac{ 2\pi }{\sqrt{ \theta _2 }}\int _{\mathbb {R}} \exp \left[ \frac{1}{2} (\theta _3y_2+\theta _4 y_2^2)^2 \sigma ^2_{1|2}(y_2) \right] \sqrt{ \sigma ^2_{1|2}(y_2)} \phi \left( y_2; 0, 1/\theta _2\right) \textrm{d}y_2 \end{aligned}$$

and hence $\eta$ is the solution to the following integral:

11 $$\begin{aligned} \eta = \log \left[ \frac{ 2\pi }{\sqrt{ \theta _2 }}\int _{\mathbb {R}} \exp \left[ \frac{1}{2} (\theta _3y_2+\theta _4 y_2^2)^2 \sigma ^2_{1|2}(y_2) \right] \sqrt{ \sigma ^2_{1|2}(y_2)} \phi \left( y_2; 0, 1/\theta _2\right) \textrm{d}y_2 \right] . \end{aligned}$$

To derive instead the conditional density of $Y_2$ given $Y_1$, it suffices to go through similar calculations as before and express the joint density as

$$\begin{aligned} f_{Y_1,Y_2}(y_1,y_2)= & {} \frac{ 2\pi \textrm{e}^{-\eta }}{\sqrt{ \theta _1 }} \exp \left[ \frac{1}{2} (\theta _3 y_1+\theta _5 y_1^2)^2 \sigma ^2_{2|1}(y_1) \right] \sqrt{ \sigma ^2_{2|1}(y_1)} \phi \left( y_1; 0, 1/\theta _1\right) \\{} & {} \quad \phi \left( y_2; \mu _{2|1}(y_1), \sigma ^2_{2|1}(y_1) \right) \end{aligned}$$

where $\sigma ^2_{2|1}(y_1)=\frac{1}{\theta _2+2\theta _6 y_1^2-2\theta _4y_1}$ and $\mu _{2|1}(y_1)=\frac{\theta _3y_1+\theta _5 y_1^2}{\theta _2+2\theta _6y_1^2-2\theta _4y_1} =(\theta _3y_1+\theta _5 y_1^2)\sigma ^2_{2|1}(y_1)$. Therefore

$$\begin{aligned} Y_2|Y_1=y_1\sim N\left( \mu _{2|1}(y_1), \sigma ^2_{2|1}(y_1) \right) \end{aligned}$$

and

$$\begin{aligned} f_{Y_1}(y_1)= \frac{ 2\pi \textrm{e}^{-\eta }}{\sqrt{ \theta _1 }} \exp \left[ \frac{1}{2} (\theta _3 y_1+\theta _5 y_1^2)^2 \sigma ^2_{2|1}(y_1) \right] \sqrt{ \sigma ^2_{2|1}(y_1)} \phi \left( y_1; 0, 1/\theta _1\right) . \end{aligned}$$

From the marginal density of $Y_1$ another possible representation for the constant $\eta$ is found:

12 $$\begin{aligned} \eta = \log \left[ \frac{ 2\pi }{\sqrt{ \theta _1 }}\int _{\mathbb {R}} \exp \left[ \frac{1}{2} (\theta _3 y_1+\theta _5 y_1^2)^2 \sigma ^2_{2|1}(y_1) \right] \sqrt{ \sigma ^2_{2|1}(y_1)} \phi \left( y_1; 0, 1/\theta _1\right) \textrm{d}y_1 \right] . \end{aligned}$$

The condition $\theta _4^2 -2\theta _2\theta _6<0$ is needed for the conditional variance $\sigma ^2_{2|1}(y_1)$ to be strictly positive $\forall y_1$.

### A.2 Proof of Proposition 2

First, we rewrite the no-arbitrage value of the contract as

13 $$\begin{aligned} \begin{aligned}{} & {} {\tilde{c}}_{t} = \textrm{e}^{-r(\tau - t)} \left( \mathbb {E}_t\left[ \left( S_{1,\tau } -K\right) I\left( S_{1,\tau }\ge \frac{a }{c}S_{2,\tau }^b\right) \right] - \mathbb {E}_t\left[ S_{2,\tau } I\left( S_{1,\tau }\ge \frac{a }{c}S_{2,\tau }^b\right) \right] \right) \text {.} \end{aligned} \end{aligned}$$

It is easy to see that the indicator appearing inside (13) equals one when $Y_2\le \frac{\sigma _1 Y_1}{b\sigma _2}-{\tilde{h}}$, with

$$\begin{aligned} {\tilde{h}} = \frac{\log \left( \frac{a S_{2,t}^{{b}} }{c S_{1,t}}\right) -\left( {\bar{r}}(1-b) + \frac{\sigma _2^2{b} -\sigma _1^2}{2} + {\lambda _2 b - \lambda _1} \right) (\tau -t)}{b \sigma _2 \sqrt{\tau -t}}\text {.} \end{aligned}$$

The first expectation is equal to

$$\begin{aligned}{} & {} \mathbb {E}_t\left[ \left( S_{1,\tau } -K\right) I\left( S_{1,\tau }\ge \frac{a }{c}S_{2,\tau }^b\right) \right] \\{} & {} \quad = \int _{\mathbb {R}} \left( S_{1,\tau }{(y_1)} - K\right) \left[ \int _{-\infty }^{ \frac{\sigma _1 y_1}{b\sigma _2}-{\tilde{h}}} f_{Y_2|Y_1}(y_2|y_1)\textrm{d}y_2 \right] f_{Y_1}(y_1) \textrm{d}y_1\\{} & {} \quad = \int _{\mathbb {R}} \left( S_{1,\tau }{(y_1)} - K \right) \varPhi \left( \frac{ \frac{\sigma _1 y_1}{b\sigma _2}-{\tilde{h}}-\mu _{2|1}(y_1)}{\sqrt{\sigma ^2_{2|1}(y_1)}}\right) f_{Y_1}(y_1) \textrm{d}y_1 \\{} & {} \quad = \int _{\mathbb {R}} \left( S_{1,\tau }{(y_1)} - K \right) \varPhi \left( {\tilde{d}}_1(y_1)\right) f_{Y_1}(y_1) \textrm{d}y_1 \text {.} \end{aligned}$$

The second expectations can be easily simplified using the following well-known result concerning the normal distribution:

$$\begin{aligned} \int _{-\infty }^a \textrm{e}^{\alpha y} \phi (y;\mu ,\sigma ^2) \textrm{d}y = \textrm{e}^{\alpha \mu +\frac{\alpha ^2\sigma ^2}{2}} \varPhi \left( \frac{a-\mu }{\sigma }-\alpha \sigma \right) . \end{aligned}$$

Therefore, by setting $\alpha _2 = \sigma _2\sqrt{\tau -t}$

$$\begin{aligned}{} & {} \mathbb {E}_t\left[ S_{2,\tau } I\left( S_{1,\tau }\ge \frac{a }{c}S_{2,\tau }^b\right) \right] \\{} & {} \quad = S_{2,t}\textrm{e}^{({\bar{r}}-\sigma _2^2/2 -{\lambda _2} )(\tau -t)} \int _{\mathbb {R}} \left[ \int _{-\infty }^{\frac{\sigma _1 y_1}{b\sigma _2}-{\tilde{h}}} \textrm{e}^{ \alpha _2 y_2} f_{Y_2|Y_1}(y_2|y_1)\textrm{d}y_2 \right] f_{Y_1}(y_1) \textrm{d}y_1\\{} & {} \quad = S_{2,t}\textrm{e}^{({\bar{r}}-\sigma _2^2/2-{\lambda _2} )(\tau -t)} \int _{\mathbb {R}} \textrm{e}^{\alpha _2\mu _{2|1}(y_1) +\frac{\alpha _2^2}{2}\sigma ^2_{2|1}(y_1) } \varPhi \left( {\tilde{d}}_1(y1)-\alpha _2\sqrt{\sigma ^2_{2|1}(y_1)} \right) f_{Y_1}(y_1) \textrm{d}y_1 . \end{aligned}$$

Putting the two expectations together gives the desired result.

## B Greeks

In this section we report the Greeks for spread options.

### Preliminaries

Write eq. (5) as ${\tilde{c}}_t = \textrm{e}^{-r(\tau -t)} \int _{\mathbb {R}} I(y)f_{Y_1}(y) \textrm{d}y$ where $I(y)= \left[ {S_{1,\tau }(y)} -K \right] \varPhi \left[ {\tilde{d}}_1(y) \right] - S_{2,t} \textrm{e}^{H(y)} \varPhi \left[ {\tilde{d}}_2(y) \right]$ and $H(y)= c_2 + \alpha _2\mu _{2|1}(y) +\frac{\alpha _2^2}{2}\sigma ^2_{2|1}(y)$. The partial derivative with respect to a quantity, *u*, appearing only in *I*(*y*), e.g. $S_{1,t}$ or $S_{2,t}$, is obtained as

$$\begin{aligned} \frac{\partial {\tilde{c}}_t}{\partial u} = \textrm{e}^{-r(\tau -t)} \int _{\mathbb {R}} \frac{\partial I(y)}{\partial u} f_{Y_1}(y) \textrm{d}y. \end{aligned}$$

The same applies to higher order partial derivatives.

*Deltas*

$$\begin{aligned}{} & {} \frac{\partial {\tilde{h}}}{\partial S_{1,t}} = - \frac{1}{S_{1,t}b \sigma _2 \sqrt{\tau -t}} \\{} & {} \quad \frac{\partial {\tilde{d}}_1(y)}{\partial S_{1,t}} = \frac{\partial {\tilde{d}}_2(y)}{\partial S_{1,t}} = -\frac{\frac{\partial {\tilde{h}}}{\partial S_{1,t}}}{\sqrt{\sigma ^2_{2|1}(y)}} = \frac{1}{S_{1,t}b \sigma _2 \sqrt{(\tau -t)\sigma ^2_{2|1}(y)}} \\{} & {} \quad \frac{\partial }{\partial S_{1,t}} \varPhi \left[ {\tilde{d}}_i(y)\right] = \frac{\partial {\tilde{d}}_1(y)}{\partial S_{1,t}} \phi \left[ {\tilde{d}}_i(y)\right] \qquad i=1,2\\{} & {} \quad \frac{\partial I(y)}{\partial S_{1,t}} = {\frac{S_{1,\tau }(y)}{ S_{1,t} }} \varPhi \left[ {\tilde{d}}_1(y)\right] +\left[ \frac{\partial {\tilde{d}}_1(y)}{\partial S_{1,t}}\right] \\{} & {} \qquad \times \left\{ [ {S_{1,\tau }(y)} -K]\phi \left[ {\tilde{d}}_1(y)\right] -S_{2,t}\textrm{e}^{H(y)}\phi \left[ {\tilde{d}}_2(y)\right] \right\} \end{aligned}$$

Assuming

$$\begin{aligned} a=S_{2,t}\textrm{e}^{{\bar{r}}(\tau -t)} +K, \qquad b=\frac{S_{2,t}\textrm{e}^{{\bar{r}}(\tau -t)}}{a}, \qquad c= {\mathbb {E}_t\left[ S_{2,\tau }^b\right] =A_1 \int _{\mathbb {R}} A_2(y) \textrm{d}y }, \end{aligned}$$

with $A_1=\textrm{e}^{ b\left[ {\log S_{2,t}+ (\tau -t)({\bar{r}}-\frac{\sigma ^2_2}{2} } {-\lambda _2} )\right] }$, and ${ A_2(y)= \textrm{e}^{ \sigma _2 b \sqrt{\tau -t} y } f_{Y_2}(y) }$, it holds that

$$\begin{aligned}{} & {} \frac{\partial b}{\partial S_{2,t}} = \frac{\textrm{e}^{{\bar{r}}(\tau -t)}\left( a-S_{2,t} {\textrm{e}^{{\bar{r}}(\tau -t)}} \right) }{a^2} \\{} & {} \quad {\frac{\partial c}{\partial S_{2,t}} = c \left[ \left( \frac{\partial b}{\partial S_{2,t}}\right) \frac{\log A_1}{b} + \left( \frac{b}{ S_{2,t}}\right) \right] + A_1 \left( \frac{\partial b}{\partial S_{2,t}}\right) \sigma _2 \sqrt{\tau -t} \int _{\mathbb {R}} y A_2(y) \textrm{d}y } \\{} & {} \quad \frac{\partial {\tilde{h}}}{\partial S_{2,t}} = \frac{ \frac{\textrm{e}^{{\bar{r}}(\tau -t)}}{a} + \left( \frac{\partial b}{\partial S_{2,t}}\right) \left( {\log S_{2,t}+ (\tau -t)({\bar{r}}-\frac{\sigma ^2_2}{2}} {-\lambda _2 )} -{\tilde{h}}\sigma _2 \sqrt{\tau -t} \right) { + \frac{b}{S_{2,t}} - \frac{\frac{\partial c}{\partial S_{2,t}}}{c}} }{b \sigma _2 \sqrt{\tau -t}} \\{} & {} \quad \frac{\partial {\tilde{d}}_1(y)}{\partial S_{2,t}} = \frac{\partial {\tilde{d}}_2(y)}{\partial S_{2,t}} = \frac{ {-} \frac{\partial {\tilde{h}}}{\partial S_{2,t}} - \frac{\sigma _1}{\sigma _2}y \frac{K \textrm{e}^{-{\bar{r}}(\tau -t)}}{S_{2,t}^2} }{\sqrt{\sigma ^2_{2|1}(y)}} \\{} & {} \quad \frac{\partial I(y)}{\partial S_{2,t}} = \left\{ [{S_{1,\tau }(y)}-K]\phi \left[ {\tilde{d}}_1(y)\right] -S_{2,t}\textrm{e}^{H(y)} \phi \left[ {\tilde{d}}_2(y)\right] \right\} \left( \frac{\partial {\tilde{d}}_1(y)}{\partial S_{2,t}} \right) -\textrm{e}^{H(y)}\varPhi \left[ {\tilde{d}}_2(y)\right] \end{aligned}$$

*Gammas*

$$\begin{aligned}{} & {} \frac{\partial ^2 {\tilde{h}}}{\partial S_{1,t}^2} = \frac{1}{S_{1,t}^2 b \sigma _2 \sqrt{\tau -t}} \\{} & {} \quad \frac{\partial ^2 {\tilde{d}}_1(y)}{\partial S_{1,t}^2} = \frac{\partial ^2 {\tilde{d}}_2(y)}{\partial S_{1,t}^2} = -\frac{\frac{\partial ^2 {\tilde{h}}}{\partial S_{1,t}^2}}{\sqrt{\sigma ^2_{2|1}(y)}} = -\frac{1}{S_{1,t}^2 b \sigma _2 \sqrt{(\tau -t)\sigma ^2_{2|1}(y)}} \\{} & {} \quad {\frac{\partial ^2 I(y)}{\partial S_{1,t}^2} } = 2\frac{S_{1,\tau }(y)}{S_{1,t}} \left( \frac{\partial {\tilde{d}}_1(y)}{\partial S_{1,t}} \right) \phi \left[ {\tilde{d}}_1(y)\right] \\{} & {} \qquad + \left( \frac{\partial ^2 {\tilde{d}}_1(y)}{\partial S_{1,t}^2}\right) \left\{ [ S_{1,\tau }(y) -K]\phi \left[ {\tilde{d}}_1(y)\right] -S_{2,t}\textrm{e}^{H(y)}\phi \left[ {\tilde{d}}_2(y)\right] \right\} \\{} & {} \qquad -\left( \frac{\partial {\tilde{d}}_1(y)}{\partial S_{1,t}}\right) ^2 \left\{ {\tilde{d}}_1(y) [ S_{1,\tau }(y) -K]\phi \left[ {\tilde{d}}_1(y)\right] -{\tilde{d}}_2(y)S_{2,t}\textrm{e}^{H(y)}\phi \left[ {\tilde{d}}_2(y)\right] \right\} \end{aligned}$$

$$\begin{aligned}{} & {} \frac{\partial ^2 b}{\partial S_{2,t}^2} = -{2}\frac{\textrm{e}^{2{\bar{r}}(\tau -t)}}{a^2} {+\frac{2S_{2,t}\textrm{e}^{3{\bar{r}}(\tau -t)}}{a^3} }\\{} & {} \quad {\frac{\partial ^2 c}{\partial S_{2,t}^2} =} \left( \frac{\partial ^2 b}{\partial S_{2,t}^2} \right) \left\{ \frac{\log A_1}{b}c + A_1 \sigma _2 \sqrt{\tau -t} \int _{\mathbb {R}} y A_2(y) \textrm{d}y \right\} \\{} & {} \qquad + \left( \frac{\partial b}{\partial S_{2,t}} \right) ^2 \left\{ -\frac{\log A_1}{b^2}c + \frac{\log A_1}{b} A_1 \sigma _2 \sqrt{\tau -t} \int _{\mathbb {R}} y A_2(y) \textrm{d}y+ A_1 \sigma _2^2 (\tau -t) \int _{\mathbb {R}} y^2 A_2(y) \textrm{d}y \right\} \\{} & {} \qquad + \left( \frac{\partial b}{\partial S_{2,t}} \right) \left\{ \frac{1}{b S_{2,t}} + \left( \frac{\partial c}{\partial S_{2,t}} \right) \frac{\log A_1}{b}+ \frac{b}{S_{2,t}} A_1 \sigma _2 \sqrt{\tau -t} \int _{\mathbb {R}} y A_2(y) \textrm{d}y \right\} \left( \frac{\partial c}{\partial S_{2,t}} \right) -\frac{c\textrm{e}^{2{\bar{r}}(\tau -t)}}{a^2}\\{} & {} \qquad + \left( \frac{\partial c}{\partial S_{2,t}} \right) \frac{b}{S_{2,t}} \\{} & {} \quad \frac{\partial ^2 {\tilde{h}}}{\partial S_{2,t}^2} = \frac{ - \frac{\textrm{e}^{2{\bar{r}}(\tau -t)}}{a^2} + \left( \frac{\partial ^2 b}{\partial S_{2,t}^2}\right) \left( {\log S_{2,t}+ (\tau -t)({\bar{r}}-\frac{\sigma ^2_2}{2}} {-\lambda _2 )} -{\tilde{h}}\sigma _2 \sqrt{\tau -t} \right) +\left( \frac{\partial b}{\partial S_{2,t}} \right) \frac{2}{ S_{2,t} } -\frac{ b}{ S_{2,t}^2} + \frac{\left( \frac{\partial c}{\partial S_{2,t}}\right) ^2 -c \left( \frac{\partial ^2 c}{\partial S_{2,t}^2}\right) }{c^2} }{b \sigma _2 \sqrt{\tau -t}} \\{} & {} \quad \frac{\partial ^2 {\tilde{d}}_1(y)}{\partial S_{2,t}^2} = \frac{\partial ^2 {\tilde{d}}_2(y)}{\partial S_{2,t}^2} = \frac{ {-} \frac{\partial ^2 {\tilde{h}}}{\partial S_{2,t}^2} + 2\frac{\sigma _1}{\sigma _2}y \frac{K \textrm{e}^{-{\bar{r}}(\tau -t)}}{S_{2,t}^3} }{\sqrt{\sigma ^2_{2|1}(y)}} \\{} & {} \quad {\frac{\partial ^2 I(y)}{\partial S_{2,t}^2}} = -2 \textrm{e}^{H(y)} \left( \frac{\partial {\tilde{d}}_1(y)}{\partial S_{2,t}} \right) \phi \left[ {\tilde{d}}_2(y)\right] \\{} & {} \qquad + \left( \frac{\partial ^2 {\tilde{d}}_1(y)}{\partial S_{2,t}^2}\right) \left\{ [ S_{1,\tau }(y) -K]\phi \left[ {\tilde{d}}_1(y)\right] -S_{2,t}\textrm{e}^{H(y)}\phi \left[ {\tilde{d}}_2(y)\right] \right\} \\{} & {} \qquad -\left( \frac{\partial {\tilde{d}}_1(y)}{\partial S_{2,t}}\right) ^2 \left\{ {\tilde{d}}_1(y) [ S_{1,\tau }(y) -K]\phi \left[ {\tilde{d}}_1(y)\right] -{\tilde{d}}_2(y)S_{2,t}\textrm{e}^{H(y)}\phi \left[ {\tilde{d}}_2(y)\right] \right\} \end{aligned}$$

*Cross Gamma*

$$\begin{aligned}{} & {} \frac{\partial ^2 {\tilde{h}}}{\partial S_{2,t}\partial S_{1,t}} = -\frac{1}{b} \left( \frac{\partial b}{\partial S_{2,t}}\right) \left( \frac{\partial {\tilde{h}}}{\partial S_{1,t}}\right) \\{} & {} \quad \frac{\partial ^2 {\tilde{d}}_1(y)}{\partial S_{2,t}\partial S_{1,t}} = \frac{\partial ^2 {\tilde{d}}_2(y)}{\partial S_{2,t}\partial S_{1,t}} = {-} \frac{1}{\sqrt{\sigma ^2_{2|1}(y)}}\frac{\partial ^2 {\tilde{h}}}{\partial S_{2,t}\partial S_{1,t}} \\{} & {} \quad {\frac{\partial ^2 I(y)}{\partial S_{2,t}\partial S_{1,t}} } = \frac{S_{1,\tau }(y)}{S_{1,t}} \left( \frac{\partial {\tilde{d}}_1(y)}{\partial S_{2,t}} \right) \phi \left[ {\tilde{d}}_1(y)\right] \\{} & {} \qquad + \left( \frac{\partial ^2 {\tilde{d}}_1(y)}{\partial S_{2,t}\partial S_{1,t}} \right) \left\{ [ S_{1,\tau }(y) -K]\phi \left[ {\tilde{d}}_1(y)\right] -S_{2,t}\textrm{e}^{H(y)}\phi \left[ {\tilde{d}}_2(y)\right] \right\} \\{} & {} \qquad - \left( \frac{\partial {\tilde{d}}_1(y)}{\partial S_{1,t}}\right) \left( \frac{\partial {\tilde{d}}_1(y)}{\partial S_{2,t}}\right) \left\{ {\tilde{d}}_1(y) [ S_{1,\tau }(y) -K]\phi \left[ {\tilde{d}}_1(y)\right] -{\tilde{d}}_2(y)S_{2,t}\textrm{e}^{H(y)}\phi \left[ {\tilde{d}}_2(y)\right] \right\} \end{aligned}$$

## Footnotes

1 See the survey of Dungey et al. (2005) for a review of methodologies on contagion.

2 Single-name option prices can be obtained quickly via one-dimensional integrals. For instance, the value of a European call option written on asset $S_i$ is

$$\begin{aligned} c_{i,t} =\textrm{e}^{-r(\tau -t)} \mathbb {E}_t\left[ \left( S_{i,\tau } - K\right) ^+\right] \end{aligned}$$

where $\mathbb {E}_t\left[ \cdot \right]$ denotes the time-*t* conditional expectation under the risk neutral probability measure. Simple calculations show that the above option price can be expressed as

$$\begin{aligned} c_{i,t} =\textrm{e}^{-r(\tau -t)} \int _{-d_{i}}^{+\infty } \left( S_{i,t} \textrm{e}^{({\bar{r}} - \frac{\sigma _i^2}{2} {-\lambda _i} )(\tau -t) + \sigma _i\sqrt{\tau -t}y} - K\right) f_{Y_i}(y) \textrm{d}y \end{aligned}$$

with $$d_{i}=\frac{\log \left( \frac{S_{i,t}}{K}\right) +({\bar{r}} - \frac{\sigma _i^2}{2}{-\lambda _i})(\tau -t) }{\sigma _i \sqrt{\tau -t}}$$.

3 For the sake of brevity, we plot only 12 out of the 231 implied density functions that we have estimated, which is roughly one trading date per month. In this sense, we view the 12 selected implied density functions as representative of our sample. However, all the estimated parameters are available from the authors upon request.

4 No arbitrage arguments required, for the purpose of option pricing, Eq. (3) to be expressed under the risk-neutral probability measure, while stocks risk-management is usually performed under the classical, or historical, probability measure. Equation (6) can be thought as the historical counterpart of (3), thus modeling the future behavior of stocks under the historical probability measure.

5 Note that, due to the symmetry of the bivariate density (1), setting $\omega =0.75$, $\theta _5=0$ and making $\theta _4$ is equivalent to the case in which $\omega =0.25$, $\theta _4=0$ and $\theta _5$ varies.
